# Differences in the Metabolic Rates of Exploited and Unexploited Fish Populations: A Signature of Recreational Fisheries Induced Evolution?

**DOI:** 10.1371/journal.pone.0128336

**Published:** 2015-06-03

**Authors:** Jan-Michael Hessenauer, Jason C. Vokoun, Cory D. Suski, Justin Davis, Robert Jacobs, Eileen O’Donnell

**Affiliations:** 1 Wildlife and Fisheries Conservation Center, Department of Natural Resources and the Environment, University of Connecticut, Storrs, Connecticut, United States of America; 2 Department of Natural Resources and Environmental Sciences, University of Illinois at Urbana-Champaign, Urbana, Illinois, United States of America; 3 Inland Fisheries Division, Connecticut Department of Energy and Environmental Protection, Marlborough, Connecticut, United States of America; Aristotle University of Thessaloniki, GREECE

## Abstract

Non-random mortality associated with commercial and recreational fisheries have the potential to cause evolutionary changes in fish populations. Inland recreational fisheries offer unique opportunities for the study of fisheries induced evolution due to the ability to replicate study systems, limited gene flow among populations, and the existence of unexploited reference populations. Experimental research has demonstrated that angling vulnerability is heritable in Largemouth Bass *Micropterus salmoides*, and is correlated with elevated resting metabolic rates (RMR) and higher fitness. However, whether such differences are present in wild populations is unclear. This study sought to quantify differences in RMR among replicated exploited and unexploited populations of Largemouth Bass. We collected age-0 Largemouth Bass from two Connecticut drinking water reservoirs unexploited by anglers for almost a century, and two exploited lakes, then transported and reared them in the same pond. Field RMR of individuals from each population was quantified using intermittent-flow respirometry. Individuals from unexploited reservoirs had a significantly higher mean RMR (6%) than individuals from exploited populations. These findings are consistent with expectations derived from artificial selection by angling on Largemouth Bass, suggesting that recreational angling may act as an evolutionary force influencing the metabolic rates of fishes in the wild. Reduced RMR as a result of fisheries induced evolution may have ecosystem level effects on energy demand, and be common in exploited recreational populations globally.

## Introduction

Recreational fishing is popular world-wide, accounting for an estimated 12% of global fish harvest [1–4]. Recreational fisheries have the capacity to interact with exploited populations in both similar and different ways than commercial fisheries [2]. Both commercial fisheries and recreational fisheries are generally size selective [5–7], typically result in higher population mortality rates [8,9], and may interrupt critical life history events [5,6,10]. However, the passive nature of recreational fishing in which a fish needs to choose to eat (or attack) the bait or lure is an important distinction [11,12]. Recreational anglers also likely have more capacity to adapt terminal tackle and presentation methods to target particular sizes of fish or to intercept particular life history stages than is common in commercial fisheries. These aspects of recreational fisheries have been shown to additionally select for behavioral phenotypes and their underlying physiological drivers [11–13] as well as morphological traits such as body shape [14]. Therefore, non-random mortality associated with commercial and recreational fisheries can produce selective responses in targeted populations, a phenomenon known as fisheries induced evolution (FIE).

Given the potential for FIE resulting from recreational angling, inland recreational fisheries offer opportunities to reveal both the presence and mechanisms of FIE. Multiple lakes and river systems can serve as replicate experimental units [15], and while rare, unexploited populations still exist [16–18]. Comparison between exploited and unexploited populations establishes a reference point for the impacts of harvest and fishing. Even in the absence of harvest, catch-and-release fisheries still have the potential to exert selective pressures as a result of unintended post-release mortality [19] and decreased reproductive fitness [20–22]. Because multiple systems can be used as replicates, gene flow is often limited among sites, and unexploited populations exist for reference, research on inland recreational fisheries associated with FIE is increasing [4,11,15,23,24].

Philipp et al. [15] demonstrated that vulnerability to recreational angling has a heritable genetic component. This was established by developing strains of Largemouth Bass (*Micropterus salmoides)* that had high vulnerability (HV) and low vulnerability (LV) to angling through an intensive multi-generation artificial selection experiment. They demonstrated that selection from angling resulted in appreciable reductions of angling vulnerability in LV fish [15], suggesting that selection towards decreasing vulnerability was feasible in exploited populations. Subsequent experiments demonstrated that relative to LV individuals, HV individuals exhibited a suite of correlated phenotypes including higher metabolic rates [23], which correlated with lower rejection rates of prey [25], and more intensive parental care and boldness [26] resulting in higher fitness potential in the absence of angling [27]. Therefore, selection from recreational fishing acts on a suite of behavioral traits linked to underlying heritable physiological processes that collectively make some individuals more vulnerable to angling [11–13]. As a result Largemouth Bass, an ecologically [28,29] and economically [30] important widespread sportfish, have emerged as a model species for study of FIE [15,20,23,26,27].

Experimental evidence suggests that recreational angling should, over time, decrease the prevalence of HV phenotypes in exploited systems leading to a decrease in overall angling vulnerability [15] and correlated phenotypes, such metabolic rates [23]. However, the phenomena described by Philipp et al. [15] may reflect an unrealistic selection regime relative to that resulting from recreational angling on wild populations. Identifying similar patterns in wild populations would not only provide needed context for the results of Philipp et al. [15], but also provide an empirical basis for future studies of recreational fisheries. If future studies were to identify FIE in many locations, managers of recreational fisheries may then need to consider evolutionary consequences of management actions similar to what is occurring in the management of commercial fisheries [31–33]. Our study provides insight into whether or not FIE may be occurring in wild populations and what magnitude of change might be expected in extant recreational fisheries.

Our objective was to examine the metabolic profiles of two exploited and unexploited populations of Largemouth Bass. We hypothesized that Largemouth Bass from exploited populations would exhibit lower metabolic rates relative to Largemouth Bass from unexploited populations, consistent with the outcomes of previous studies that used Largemouth Bass line-bred for differences in angling vulnerability [15,23].

## Materials and Methods

### Ethical Statement

Largemouth Bass are not endangered or protected in the State of Connecticut where sampling occurred. Our animal use and care protocols were approved by the University of Connecticut Office of Research Compliance Institutional Animal Care and Use Committee, protocol A12-012. Sampling permission was granted under a scientific collectors permit issued by the Connecticut Department of Energy and Environmental Protection Inland Fisheries Division. Permission for sampling at Hemlock Reservoir and Easton Reservoir was granted by the Aquarion Water Company.

### Sampling Methods

We collected Largemouth Bass from four populations in Connecticut, USA, during fall 2012. Hemlock Reservoir (177 ha; N 41°13’2.27”, W 73°17’19.78”) and Easton Reservoir (198 ha; N 41°15’55.25”, W 73°16’1.10”) are private drinking water reservoirs and were impounded in 1914 and 1926, respectively, by the Bridgeport Hydraulic Company. Both reservoirs have been closed to angling since their construction. While some illegal shoreline angling likely occurs, both of these lakes lack public boat access and are patrolled by water company law enforcement officers; resulting in populations that have essentially been unexploited by anglers for almost a century [16]. In contrast, both Amos Lake (46 ha; N 41°31’1.57”, W 71°58’31.14”) and Gardner Lake (214 ha; N 41°30’39.66”, W 73°13’38.77”) support popular public Largemouth Bass fisheries, have public boat ramps, and were therefore deemed ‘exploited’ for the purposes of this study. All four lakes are mesotrophic, and Largemouth Bass are the dominant piscivore. Fish communities consist of Centrarchidae (primarily Bluegill *Lempomis macrochirus* and Pumpkinseed *Lepomis gibbous*) and Percidae (primarily Yellow Perch *Perca flavescens*) species. Gardner and Amos Lake are seasonally stocked with Brown Trout *Salmo trutta* and Rainbow Trout *Oncorhynchus mykiss* as part of put-and-take fisheries practices, and while not stocked directly Hemlock and Easton Reservoirs contain Brown and Rainbow trout from the downstream displacement of fish stocked upstream. Hemlock Reservoir and Amos Lake also contain populations of landlocked Alewives *Alosa pseudoharengus*. Largemouth Bass are not native to Connecticut, and are believed to have been introduced throughout the state (and region) from an upstate New York source population via Federal stocking programs in the 1850’s [34]. It has not been management practice to stock Largemouth Bass in any lakes or reservoirs in the state since early naturalization, and no records of such stockings exist for the study lakes. All four systems experience the same climate conditions, given their close latitude and proximity to Long Island Sound.

Age-0 Largemouth Bass were captured from widely distributed locations within each source lake via beach seining, trap netting, and boat electrofishing. Sampling occurred over approximately four weeks beginning September 25^th^ 2012 and ending October 24^th^ 2012. Sampling effort was consistent among the three gears in each lake. Trap netting entailed setting five trap nets over 48 h intervals, with each trap emptied each morning. Seining occurred over a two work day period (concurrent with trap netting) and night-time electrofishing completed one full lap of each lake. We chose to sample each lake with a combination of active (seining, and electrofishing) and passive (trap-netting) techniques employed with similar effort among lakes because vulnerability to sampling gears has been shown to vary with animal personality e.g. [35–37]. Animal personality has been linked to metabolic rate [38], therefore our sampling approach was designed to guard against collecting an unrepresentative sample of available personality traits and metabolic rates. Age-0 Largemouth Bass disperse widely and randomly from their nest of origin [39], suggesting that our sample is unlikely to be biased towards a small number of kin groups, and thus confounded by potential maternal effects within populations. After capture, Largemouth Bass were transported 107, 92, 83, and 95 km from Amos, Easton, Gardner, and Hemlock, respectively, in aerated coolers to a single outdoor pond at the Connecticut Department of Energy and Environmental Protection Burlington State Fish Hatchery in Burlington, Connecticut. Prior to release into the pond, individuals from each population were double tagged with population-specific batch marks including a fin clip and visual implant elastomer tag. Throughout spring and summer of 2013, the pond was stocked at regular intervals with small minnows for forage (*Pimephales promelas* and *Notemigonus crysoleucas*).

During fall 2013, after individuals had acclimated and been reared in the common pond for at least 10 months, we measured field resting metabolic rates (RMR) of individuals from each of the four populations. Each day, four individuals of similar size (one from each source population) were moved to a closed pen immersed in the pond and held without food for 48 h. After this 48 h period, fish were randomly assigned to respirometry chambers to measure their oxygen consumption using intermittent, flow-through respirometry [40]. The respirometry system, housed inside a 1.83 × 3.05 × 1.83 m cargo trailer parked at the side of the pond, was identical to the system used by Redpath et al. [23]. The respirometer contained four chambers (~0.75 L total volume) immersed in a 340 L water tank. Water in the tank was aerated continuously and exchanged daily with pond water. Tank water temperatures were initially matched to pond temperatures at the time fish were placed in the respirometry chambers (mean temperature 18.5 C; range 15.0–20.4 C), and then held constant throughout each trial using a digitally controlled 250 W aquarium heater. Water flow in each chamber was controlled by two aquarium pumps. The first pump circulated water through the chamber past a fiber optic oxygen probe connected to an OXY-4 fiber optic oxygen instrument (Loligo Systems, Tjele, Denmark), which measured variation in oxygen partial pressure. The second pump exchanged water within the respirometry chamber with aerated tank water. Pumps were automatically controlled by the AutoResp 4 software (Loligo Systems, Tjele, Denmark), which alternated between a four minute flush phase, a one minute wait phase and an 8–20 minute measurement phase (duration selected by the authors based on fish size and water temperature). The same measurement phase was applied to all four fish during a given trial, such that overall measurement lengths were not different among the four populations. Oxygen partial pressure was measured twice per second and regressed against time. The slope of the regression line comparing oxygen concentration over time was used to calculate oxygen consumption using the equation:

$$Mo_{2}=\frac{k\times V\times \alpha }{m}$$

where Mo_2_ is oxygen consumption (mg O_2_·kg^-1^·h^-1^), k is the slope of the regression line, V is the volume (in L) of water within the chamber (corrected for fish volume), α is the solubility of oxygen in water at the experimental temperature, and m is the mass (kg) of the fish.

Trials were initiated during the afternoon, and individuals were left undisturbed overnight in their randomly assigned chambers, resulting in a total trial length of 20–22 h. This procedure generated one data point for each fish every 13–25 min, for a total of 47–100 data points per trial. To ensure high quality data, we limited analysis to data points generated from measurement phases resulting in oxygen consumption over time slopes with regression r^2^ > 0.90 [41,42]. For each individual we also examined a graph of oxygen consumption over total trial time to ensure that fish were not becoming active during a given measurement phase (which would manifest as a spike in oxygen consumption). Any fish that became active during multiple measurements were excluded from the dataset. After removing fish from the chambers, background oxygen consumption rates were quantified by resuming the collection of data using empty chambers for approximately 90 minutes, generating six blank measurements. All equipment was sterilized and sensors were calibrated regularly throughout the data collection.

Field RMR for each individual was defined as the six lowest observations per individual from the 47–100 data points collected during the trial, corrected for mean background oxygen consumption [43,44]. Log transformed field RMR was normally distributed (Shapiro Wilk W = 0.99, P = 0.84). Fish weights (Amos N = 30: 33 ±1 g, Easton N = 25: 32 ± 2 g, Gardner N = 25: 33 ± 2 g, Hemlock N = 31: 31 ± 1 g; ANOVA: F_3,107_ = 0.69, P = 0.56) and lengths (Amos: 141 + 2; Easton: 139 + 2; Gardner: 140 + 2; Hemlock: 135 + 2; ANOVA: F3,107 = 1.47, P = 0.22) did not differ significantly among populations, hence fish weight and length were excluded from further analysis. The effect of exploitation status (exploited vs. unexploited) on log field RMR was evaluated using a mixed model analysis of covariance (ANCOVA) [45]. For the ANCOVA, log temperature, exploitation status, population of origin (nested within exploitation status), as well as the interaction between temperature and population of origin were treated as fixed effects. Fish identification number, respirometry tube number, and trial day were treated as random effects. This approach accounts for differences in temperature among respirometry trials and allows the variability among individuals, populations, and day of the trial to be explicitly included within the model [44,46]. Model residuals were normally distributed (assessed by examining the normal quantile plot), and mean residuals were not significantly different among the four populations, indicating that the assumptions of the model were met. The distribution of mean RMR corrected to mean temperature (18.5 C) across all trials based on the temperature vs. metabolic rate relationship defined by the ANCOVA analysis among unexploited and exploited individuals were compared using a Kolmogorov-Smirnov (KS) test. Statistical analysis was conducted in JMP 11 (mixed-modeling, and residual analysis) and Program R 2.13.2 (KS test). Results were considered statistically significant when P ≤ 0.05.

Finally, we performed a simplistic modeling exercise to illustrate the potential effects of different RMR for exploited and unexploited populations on ecosystem trophic dynamics. Two populations (one exploited and one unexploited) of 1,000 adult individuals were modeled, with a mean weight of 0.9 kg per individual. We limited the exercise to a growing season of 200 d, with a water temperature of 25°C. We used the oxy-caloric multiplier of 3,800 cal·g^-1^ O_2_ [47] to convert field RMR into calories used by each population. We assigned adults from the exploited population a RMR of 0.102 g O_2_·kg^-1^·h^-1^ [48], and unexploited individuals a RMR 6% higher (see results). Outcomes of this exercise were expressed in kilograms of two common prey species, Bluegill (*Lepomis macrochirus*) and Fathead Minnows (*Pimephales promelas)* based on the caloric density of those species.

## Results and Discussion

**Fig 1:**
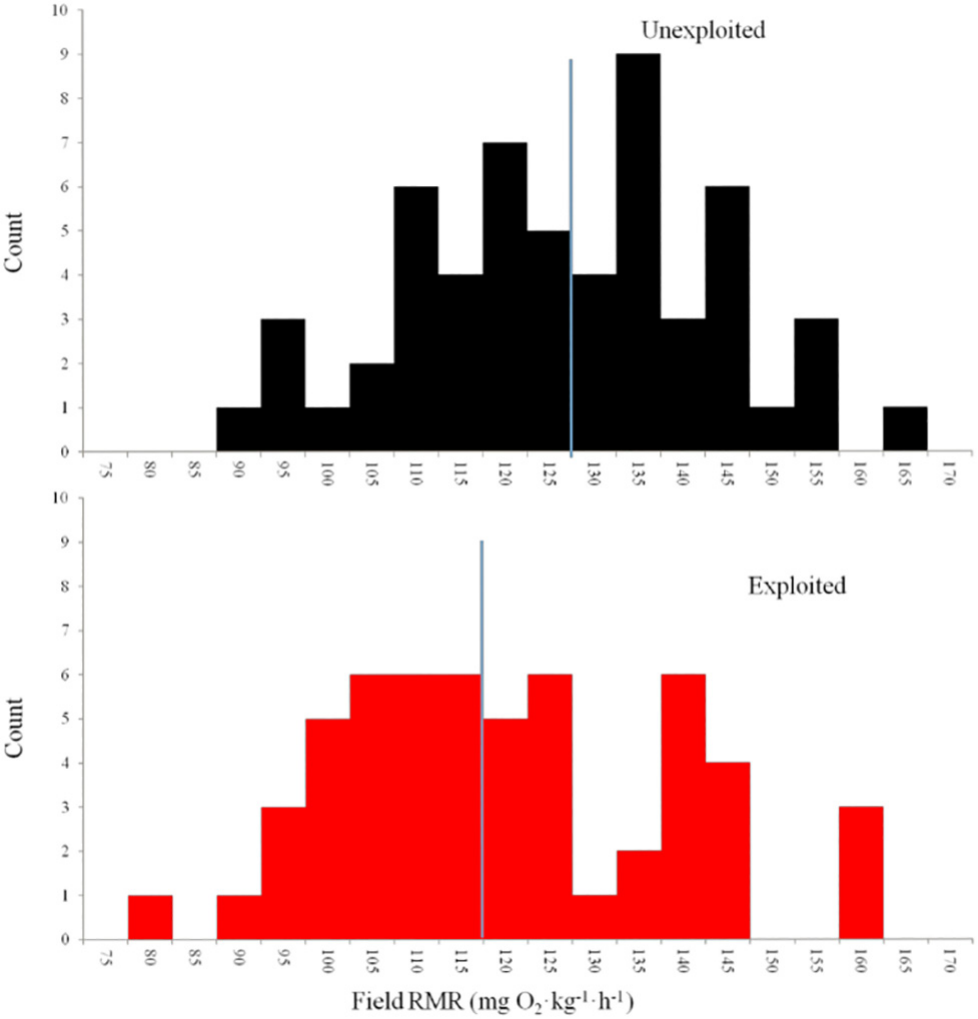
Distribution of Field Resting Metabolic Rates. Histogram of field resting metabolic rates of unexploited populations (black bars) and exploited populations (red bars) of Largemouth Bass raised in a common environment. X-axis values represent the starting value of each bin. Vertical reference line represents the median of each population.

**Fig 2:**
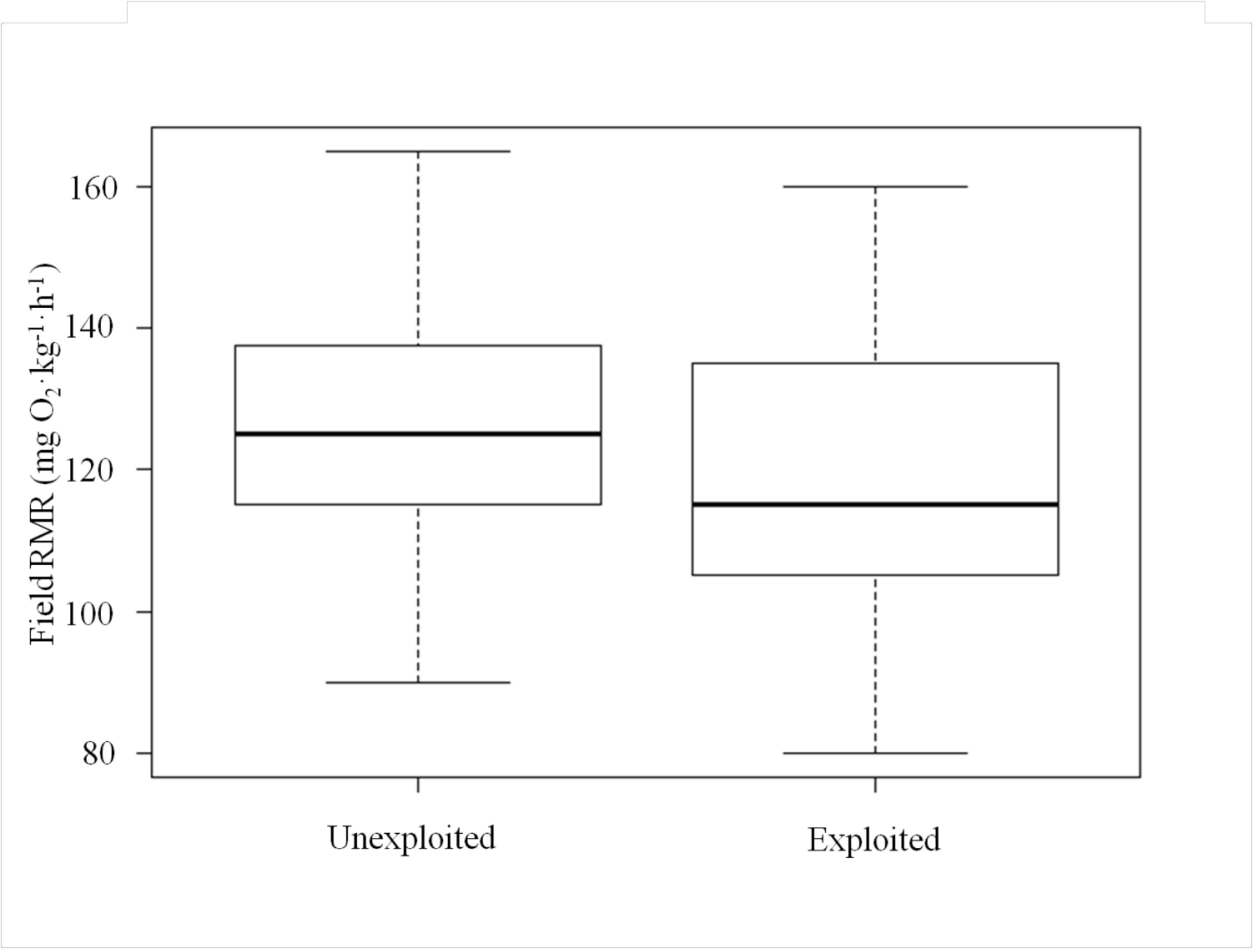
Boxplot of Field Resting Metabolic Rates. Boxplot of field resting metabolic rates of unexploited and exploited populations of Largemouth Bass raised in a common environment. The horizontal black line represents the population median, the top and bottom of each box represent the 75^th^ and 25^th^ quartiles respectively.

The potential for recreational angling to act as an evolutionary force is well established in theory [4,12,14,15,27], and this study represents a first step towards identifying outcomes of selection from angling in wild recreationally-targeted populations using unexploited populations as references. In our study mean field RMR of largemouth bass from unexploited reservoirs was approximately 6% higher than that of fish from lakes open to fishing (unexploited least squares mean: 118.2 mg O_2_·kg^-1^·h^-1,^ exploited least squares mean: 111.6 mg O_2_·kg^-1^·h^-1^; F_1,73_ = 8.34, P = 0.005, Table 1). In addition, the temperature-corrected and back-transformed range of observations was similar for both exploited and unexploited populations (unexploited range: 86.5–163.1 mg O_2_·kg^-1^·h^-1^, exploited range: 79.9–156.3 mg O_2_·kg^-1^·h^-1^; Fig 1). However, the groups had significantly different distributions (D = 0.26, P = 0.04) where 75% of unexploited individuals had higher metabolic rates than the median of the exploited populations (exploited population median: 113.2 mg O_2_·kg^-1^·h^-1^; Fig 2), and 71% of exploited individuals had metabolic rates lower than the median of the unexploited populations (unexploited population median: 124.8 mg O_2_·kg^-1^·h^-1^). While our study cannot eliminate all alternative explanations (we discuss some potentials below), we did document clearly the expected pattern of FIE (following Philipp et al. [15] and Redpath et al. [23]) in replicate wild fish populations subject to recreational fisheries. Because our results align with previous predictions by Philipp et al. [15] we hypothesize that recreational angling was the likely agent of selection driving metabolic differences among exploited and unexploited populations of wild Largemouth Bass examined in this study.

**Table 1:** Mixed Model ANCOVA Table.

Source	DF	Dfden	F	P
Temperature	1	444	118.8	<0.001
Exploitation Status	1	73	8.344	0.005
Population (Exp Stat)	2	75	1.479	0.235
Pop*Temp	3	326	0.773	0.510

Table of main effects for the analysis of covariance (ANCOVA), quantifying the effects of temperature, exploitation status and population on the resting metabolic rate of 4 populations of Largemouth Bass. Population was modeled nested within exploitation status to account for the fact that there were two populations each from the exploited and unexploited treatments.

For black bass (*Micropterus spp*.), selection due to angling is known to influence a range of behaviors including foraging [25] and reproduction [27]. Angling during the vulnerable nesting stage has negative effects on the fitness of individual nest-guarding males by decreasing nest success even when removal is temporary (e.g., catch-and-release) [20,22,49]. Likewise, increased mortality rates associated with angling (e.g., purposeful harvest or post-release hooking mortality) may have selective effects on exploited populations [9]. Therefore, the mechanism for FIE exists given that recreational angling preferentially targets individuals with a collection of heritable phenotypes, including boldness, foraging behavior, and nest defense that collectively increase angling vulnerability [27]. Previous studies suggest that Largemouth Bass populations may be evolving (or may have already evolved) towards decreased vulnerability to angling [15]. Our finding, that unexploited populations contained more individuals with higher metabolic rates than those from exploited populations, was predicted by the results obtained using Largemouth Bass bred for high and low angling vulnerability [23], and supports the hypothesis that angling may be altering population metabolic rates. Yet the continued presence of high metabolic rate phenotypes at low abundance in exploited populations suggests that if selective effects of angling are occurring, mitigation may be possible. Sutter et al. [27] found that high metabolic rate individuals had higher reproductive fitness than those with low metabolic rates. If one assumes that wild, high metabolic rate individuals also have higher fitness relative to wild low metabolic rate individuals in the absence of angling, then restricting angling during the nesting season may promote the fitness of high metabolic rate phenotypes and increase their abundance in the population. Exploring the mechanisms through which high metabolic rate phenotypes are retained in exploited populations has conservation importance, especially if future studies confirm that FIE in recreational fisheries is widespread. One explanation may be that selection has not been sufficiently strong, or occurred for a sufficient time period, to eliminate these phenotypes. Alternatively, the fitness advantage for high metabolic rate phenotypes [27] when not captured by anglers may be sufficient to maintain these phenotypes in the population.

The disruption of food web structures may limit the recovery of exploited fish stocks [24,50] and our results infer that different basal prey demand may exist between unexploited and exploited populations. We performed a simple exercise to evaluate how a 6% difference in Largemouth Bass RMR and resultant moderation of top-down predation might influence ecosystem-level trophic dynamics. Extrapolation of field RMR to a population for an entire year is difficult because of variations associated with water temperature, individual sizes, etc.; however, we assumed that the 6% difference in RMR was maintained between exploited and unexploited individuals. This assumption requires further testing, but we make it here because the four populations we examined responded similarly to temperature. Based solely on basal metabolic demand 1,000 unexploited individuals would require 100,600,000 more calories during a 200 day season than 1,000 exploited individuals. This caloric demand is equivalent to the caloric content of 86.7 kg of Bluegill (*Lepomis macrochirus*) [51] or 142.5 kg of Fathead Minnow (*Pimephales promelas)* [52]. The effects that such energetic differences among exploited and unexploited individuals have on a systems trophic dynamics would of course be modulated by system productivity and other factors. While this exercise is too simplistic to be quantitatively predictive, it nonetheless suggests that a 6% difference in RMR could affect biomass at lower trophic levels, thus having substantial energetic consequences at the ecosystem level.

An alternative explanation for the observed results is that unmeasured environmental variation among the four study lakes could have driven the differences in RMR observed. Subtle differences in climatic conditions influencing the exploited and unexploited reservoirs might explain the differences in metabolic rates that we observed. All four systems are in relatively close proximity to Long Island Sound which has strong effects on local climate conditions, but the possibility of subtle differences which could impact the systems remains. We statistically tested for differences in the four populations studied here and found that population of origin was not a significant predictor of field RMR, once the effect of exploitation status had been accounted for (F_2,75_ = 1.48, P = 0.23). This finding suggests if some unmeasured environmental variation were confounding our results, it would have been distributed among the four populations in the same manner as exploitation status (i.e., similar in two lakes and the same, but different in the other two). Not surprisingly, field RMR increased with temperature (F_1,444_ = 118.8, P < 0.001), however, the interaction between temperature and population of origin was also not significant (F_3,326_ = 0.77, P = 0.51). If climatic conditions, such as temperature regime, were driving differences in metabolic rates among the study populations we hypothesized that a significant difference in the population by temperature interaction would be observed, when fish were acclimated to a common environment. The lack of a significant population by temperature interaction is one indication that all four populations responded similarly to the temperature range (15.0–20.4 C) experienced during data collection, but we cannot equivocally rule out the possibility that unmeasured differences in climatic conditions affected metabolic rates among the source populations.

Also, Hemlock and Easton Reservoirs had higher densities of Largemouth Bass than another exploited Connecticut population of Largemouth Bass [16], but interestingly, the condition (mass-at-length) of adult fish did not differ among exploited and unexploited populations in that study [16]. It has been shown in mammals that net primary productivity of different environments (i.e., desert versus woodland) can lead to differences in basal metabolic rates among related species [53], in which those with ample food tend to ‘idle fast’. However, the differences in fish density among the four source populations were most likely related to exploitation status, given the physiographic proximity and general similarity of the waterbodies, and may present an alternate mechanism of how fishing could alter metabolic rates among exploited and unexploited populations such that a reduction in density from fishing could ‘release’ primary production and promote higher metabolic rates. However, in the present study higher density unexploited populations had higher metabolic rates than exploited populations. Therefore, our findings are more congruent with the mechanism of selection from angling on a suite of correlated behavioral phenotypes and their underlying physiological processes [11–13,15,23].

Maternal effects are another possible explanation of the differences that we observed in RMR. Research has indicated that female Three-Spined Stickleback (*Gasterosteus aculeatus*) can transfer environmental information via their eggs, resulting in higher metabolic rates in high predation (stressful) environments [54]. In the current study angling represents an additional predation stress which differed among parents of our test subjects, and the finding of higher metabolic rates in unexploited populations run contrary to those observed in stickleback. Maternal effects, classically controlled, require breeding in a common environment and testing of F2 individuals. Such an experiment would be needed to determine whether maternal effects could explain some or all of the differences in RMR observed. However, such an experiment would be difficult to implement with a large sample of Largemouth Bass and researchers would need to carefully monitor the influence of unwanted selection (e.g., adaptation to captivity [55]) given the size at maturity and the reproductive biology of Largemouth Bass.

The current study represents a snapshot of differences among two unexploited and two exploited populations of Largemouth Bass. Changes in angler behavior may alter the strength and perhaps direction of selection on Largemouth Bass populations. Recently, many recreational fisheries in North America have experienced a shift towards catch-and-release practices rather than harvest, where large portions of fish (approaching 100% in some cases) are released following capture [4]. If one assumes that harvest-oriented recreational fisheries exert greater (or different) selective forces than catch-and-release angling, then selection on traits that are potentially affected by fishing may be changing. For example, catch-and-release practices may have relaxed selection on boldness or foraging behaviors by reducing the probability of mortality associated with angling. However, as mentioned above, catch-and-release angling has potential to cause selective effects on individuals through the disruption of reproductive behavior [20,22,49], or unintentional post-release mortality. Given that high metabolic rate phenotypes are known to be more vulnerable to angling [23], future studies should seek to quantify the metabolic rates of individuals originating from an unexploited source population exposed to various angling treatments (e.g., catch-and-release, various levels of annual harvest, no angling during spawn, etc.) and intensities over multiple generations. Such a study would inform scientists about the relative strength of selection of different angler behaviors. Continued monitoring of these populations after angling ceased could be used to evaluate recovery rates. Such an experiment could reveal how likely changes in management would be to elicit population-level phenotype recovery.

In summary, comparisons to unexploited reference populations facilitated a straightforward detection of RMR differences consistent with the predictions of FIE in wild exploited Largemouth Bass populations. Future study should involve the controlled breeding of individuals from unexploited reference populations, and could conclusively determine that the patterns that we observed resulted from selection by angling alone. If FIE is occurring it is likely that similar differences could be found throughout the range of Largemouth Bass, and similar outcomes may be expected in other marine and inland recreational fisheries, especially if individuals exhibit variation in behavior that makes some more vulnerable to angling relative to others. Eikeset et al. [32] presented a model developed for Atlantic Cod (*Gadus morhua*) that predicted evolutionary change, even with low fishing mortality, and concluded that management to avoid FIE was unfeasible. Establishing the extent to which FIE has occurred or is occurring in populations subject to recreational fishing, and the patterns of change in population-level distributions of phenotypes associated with FIE seems the next step. Finally, learning whether or not these changes are altering trophic dynamics or represent a problem for fisheries sustainability and quality is critical for the future management of recreational fisheries and the aquatic ecosystems within which they reside.
